# Recent advances in antiviral interferon-stimulated gene biology

**DOI:** 10.12688/f1000research.12450.1

**Published:** 2018-03-12

**Authors:** John W. Schoggins

**Affiliations:** 1 Department of Microbiology, University of Texas Southwestern Medical Center, Dallas, TX, 75390, USA

**Keywords:** antiviral interferon-stimulated genes, viral pathogenesis, interferon response

## Abstract

The interferon response protects cells from invading viral pathogens by transcriptionally inducing the expression of interferon-stimulated genes (ISGs), some of which encode effectors with varied antiviral functions. As screening technologies improve and mouse model development quickens, more ISGs are continually being identified, characterized mechanistically, and evaluated for protective roles *in vivo*. This review highlights selected recent findings of ISG effectors that contribute to our understanding of the interferon antiviral response.

## Introduction

A major component of cell-intrinsic antiviral defense in higher eukaryotes is the interferon (IFN) response. All mammalian IFNs have some capacity to suppress viral replication, and type I and III IFNs are considered the main antiviral cytokines. IFNs activate JAK–STAT signaling, which leads to the transcriptional induction of hundreds of IFN-stimulated genes (ISGs). The ISG-encoded proteins include direct effectors which inhibit viral infection through diverse mechanisms as well as factors that promote adaptive immune responses. Notably, many molecules that positively or negatively regulate IFN production are themselves ISGs. Examples include IFN regulatory factors (IRF7 and IRF1), viral sensors (RIG-I, MDA5, and cGAS), and negative regulators (USP18 and SOCS1). In the field of cell-intrinsic antiviral immunity, there is intense interest in uncovering the identity of antiviral ISGs and characterizing their mechanisms of action and roles in viral pathogenesis.

In this brief review, I aim to cover recent advances in our understanding of antiviral ISG biology and highlights from the last 2–3 years in three specific areas: (1) new molecular insight into previously characterized antiviral ISGs, (2) new roles of previously characterized ISGs in viral pathogenesis, and (3) identification of newly discovered antiviral ISG effectors. As more ISGs are found to inhibit an increasing number of viruses across numerous animal species, it is not possible to summarize every report of all ISG-virus combinations. Rather, this review will limit discussion to a few select new or known antiviral ISGs for which significant mechanistic insight or biological relevance has been obtained in recent years.

## New molecular insight into previously characterized antiviral interferon-stimulated genes

### Interferon-induced proteins with tetratricopeptide repeats family

The interferon-induced proteins with tetratricopeptide repeats (IFIT) family members have been shown to broadly inhibit multiple viruses through translation inhibition. One member of the family, mouse Ifit1, was previously shown to specifically target viral RNAs lacking 2′O-methylation, known as a cap(0) structure ^1, 2^. Since mammalian RNAs are normally 2′O-methylated to give a cap(1) structure, Ifit1 has been proposed to distinguish self from non-self RNA. However, a curious finding in the field was that human IFIT1 did not possess the same antiviral properties as murine Ifit1. In a recent study on the IFIT family, Daugherty *et al*. explored the mechanism underlying this functional discrepancy ^3^. Using a robust phylogenetic analysis pipeline, the authors found that members of the IFIT family can be grouped into five distinct gene families: *IFIT1*, *IFIT1B*, *IFIT2*, *IFIT3*, and *IFIT5*. Mouse Ifit1 is more appropriately placed in the *IFIT1B* family, making it a paralog to human *IFIT1* but not an ortholog. Indeed, in a functional growth assay in budding yeast, in which RNAs contain only cap(0) structures, IFIT1B from multiple species suppressed yeast growth in a cap(0)-dependent manner whereas the *IFIT1* family members did not. However, the *IFIT1* family members did suppress yeast growth in this assay in a manner independent of cap(0) structures, suggesting a distinct mechanism of translation inhibition. In functional viral infection assays in mammalian cells, the authors further delineated the non-overlapping antiviral specificity of IFIT1 and IFIT1B for different viruses. They confirmed that IFIT1B suppressed the translation of viruses with cap(0) but not cap(1) structures. IFIT1 proteins were not effective against viruses bearing cap(0) structures. IFIT1 did, however, suppress vesicular stomatitis virus, which bears a cap(1) structure. Combined with the yeast growth assay, these viral specificity experiments suggest that the IFIT1 proteins may also distinguish self from non-self RNA through an unknown molecular pattern.

In another study on the role of murine Ifit1, now Ifit1b, alphaviruses were found to exhibit differential sensitivity to Ifit1b-mediated inhibition ^4^. Mechanistically, this regulation was associated with specific stem-loop structures in the 5′ terminus of viral genomic RNA. The authors propose that sequence variation in the stem-loop structure can alter the binding affinity of viral RNA for Ifit1b, thereby modulating sensitivity to Ifit1-mediated inhibition of viral translation.

### Viperin

The gene *RSAD2* encodes the antiviral protein viperin, which is one of the most highly induced ISGs. Viperin is a member of the vast family of radical *S*-adenosylmethionine (SAM) enzymes, which use a [4Fe-4S] cluster to cleave SAM, thereby initiating diverse radical reactions. Viperin has been shown to have broad antiviral activity against numerous RNA and DNA viruses through seemingly distinct phases of the viral life cycle. For example, it targets genome replication of several flaviviruses and egress of influenza A virus. For some viruses, the radical SAM activity is thought to be dispensable, but it is required for the inhibition of other viruses. Although the exact molecular mechanism underlying the antiviral activity of viperin is still unknown, several recent studies have revealed new biochemical and cell biological insight into this protein.

Though viperin was first reported 18 years ago ^5^, its crystal structure has only recently been solved ^6^. Fenwick *et al*. anaerobically purified a fragment of mouse viperin either in complex with the SAM analog *S*-adenosylhomocysteine or in complex with 5′-deoxyadenosine and L-methionine, both products of SAM cleavage ^6^. Their data indicate that viperin contains a conventional radical SAM enzyme fold, in which the conserved CX3CX2C motif binds the [4Fe-4S] cluster. Similar structural features were shown between viperin and another radical SAM enzyme, molybdenum cofactor biosynthetic enzyme MoaA. Based on analyses of the active site, the authors propose that viperin may use nucleoside triphosphate as a substrate. These structural data were complemented by cell culture studies demonstrating that viperin interacts with multiple members of the cytosolic iron-sulfur protein assembly machinery ^7^. Shortly after these publications, Honarmand Ebrahimi *et al*. reported on the catalytic activity of a fungal viperin ortholog ^8^. The authors hypothesized that, based on its localization to the cytosolic side of the endoplasmic reticulum and known antiviral roles, viperin may use nucleotide sugars as substrates. Indeed, the authors found that uridine diphosphate (UDP)-glucose is a substrate for purified viperin. Docking fungal viperin onto the published structure of mouse viperin revealed a conserved binding pocket for UDP-glucose. Whether this *in vitro* substrate and catalytic mechanism are linked to antiviral activity in the cell remains to be determined.

## New roles of previously characterized interferon-stimulated genes in viral pathogenesis

### Interferon-inducible transmembrane family

The interferon-inducible transmembrane (IFITM) proteins inhibit at least 12 diverse enveloped viruses, primarily by targeting viral entry ^9, 10^. A role for IFITM3 in viral pathogenesis *in vivo* has been demonstrated in the context of influenza A virus and respiratory syncytial virus but not other viruses. Two recent studies demonstrated that Ifitm3 also controls alphavirus and flavivirus infection and pathogenesis in mice ^11, 12^. Poddar *et al*. showed that deletion of murine *Ifitm3* only or the entire *Ifitm* locus resulted in higher levels of alphavirus replication in cultured mouse embryonic fibroblasts ^11, 12^. *In vivo*, *Ifitm3* ^−/−^ mice were more susceptible to chikungunya infection and exhibited higher degrees of arthritogenic outcomes relative to control mice. Similarly, *Ifitm3* ^−/−^ mice challenged with Venezuelan equine encephalitis virus were more susceptible to lethal viral infection and had elevated levels of viral titers in liver, spleen, and central nervous system tissues. In a mouse model of West Nile virus infection, Gorman *et al*. showed that Ifitm3 primarily suppressed viral replication in non-neuronal cells and that, in its absence, mice were vulnerable to lethal infection ^12^. In a separate study, Ifitm3 was shown to control murine cytomegalovirus pathogenesis but did so independently of cell-intrinsic inhibition of viral replication ^13^. Rather, in this viral model, loss of Ifitm3 resulted in impaired cytokine production, which led to the loss of critical immune cells (natural killer and T cells) that control viral replication. Similarly, IFITM3 was also implicated in regulating the ability of cells to produce IFN upon Sendai virus infection ^14^. Together, these recent studies expand the repertoire of IFITM3 function and the mechanisms involved in IFITM3-mediated restriction of viral pathogenesis *in vivo*.

### IFI-6-16 family

Another ISG family is the IFI-6-16 family, which consists of IFI6, IFI27, IFI27L1, and IFI27L2 in humans and IFI27, IFI27L2A, and IFI27L2B in mice. Several studies have implicated Ifi27 and Ifi27L2a in controlling viral infection ^15, 16^. A recent study further explored the antiviral properties of Ifi27L2a on West Nile virus pathogenesis *in vivo* ^17^. *Ifi27L2a* ^−/−^ mice were more susceptible to lethal West Nile virus infection, which was associated with higher viral titers and altered neuronal cell death patterns in specific brain regions. Although the specific mechanism of IFI27L2A-mediated protection is still unclear, the antiviral activity of IFI27L2A, like other members of this family, may be linked to cell death phenotypes in specific organs or tissues. For the human IFI-6-16 family member IFI6, *in vivo* data are still lacking, particularly since mice do not have an IFI6 ortholog. However, several recent *in vitro* studies on IFI6 have been reported but with conflicting results. Whereas one study reported that IFI6 enhances hepatitis C virus (HCV) replication ^18^, another has suggested that IFI6 inhibits HCV infection ^19^. A third study implicates HCV p7 as an immune evasion protein that antagonizes IFI6-mediated inhibition ^20^. The phenotypic differences in these studies are substantial, indicating that additional work is needed to reconcile the role of IFI6 in HCV infection.

### Cholesterol-25-hyroxylase

Cholesterol-25-hyroxylase (CH25H) is an antiviral ISG effector that catalyzes the formation of 25-hydroxycholesterol (25HC) from cholesterol. In several studies, 25HC has been shown to suppress viral infection by pathogenic viruses, including vesicular stomatitis virus, HIV-1, and Ebola virus ^21, 22^. In a recent study, the repertoire of CH25H-targeted viruses was extended to Zika virus ^23^. Li *et al*. used multiple approaches to demonstrate that 25HC broadly inhibits Zika virus and other flaviviruses by blocking viral entry ^23^. Treatment of mice and rhesus monkeys with 25HC suppressed Zika virus viremia. Furthermore, the authors showed that 25HC can suppress Zika infection in human cortical neuron organoids as well as limit infection and in a mouse model of Zika-associated microcephaly. These studies demonstrate that 25HC, the soluble product of the ISG CH25H, has *in vivo* protective effects against pathogenic outcomes of Zika virus infection.

## Identification of newly discovered antiviral interferon-stimulated gene effectors

### C19orf66/RyDEN/IRAV

In a cDNA library-based screen for IFN-induced transcripts that suppress dengue virus, Suzuki *et al*. uncovered human C19orf66, which they named RyDEN, as a potent inhibitor of dengue virus replication ^24^. Using affinity purification–mass spectrometry analysis, the authors reported that C19orf66 interacted with several RNA-binding proteins, including PABPC1 and LARP1, and proposed that its effector function likely targeted the translation of viral RNA. They also found that several other viruses were inhibited by C19orf66, suggesting a broad antiviral function. Another group found that C19orf66, which they called IRAV, localized near MOV10 in cytoplasmic processing bodies (P bodies) ^25^. Upon infection with dengue virus, C19orf66 localized near viral replication complexes, where it was found to co-immunoprecipitate with viral proteins. In a preprint study that has yet to be peer-reviewed at the time of writing, C19orf66 was also shown to inhibit the production of HIV-1 ^26^. This result is consistent with earlier findings that ectopic expression of C19orf66 did not support robust production of replication-defective lentivirus reporter vectors ^27^. Together, multiple lines of evidence suggest that C19orf66 may be an important new antiviral effector, although more insight into its mechanism of action is needed.

### ADAP2

A microarray analysis of human fibrosarcoma cells identified ArfGAP with dual pleckstrin homology domains 2 (ADAP2) as an ISG that restricts dengue virus and vesicular stomatitis virus ^28^. ADAP2 was shown to promote macropinocytosis in an Arf6-dependent manner, suggesting that its antiviral effect may be due to diverting viruses from a productive entry pathway. Notably, ADAP2-mediated inhibition of viral infection still occurred after mitochondrial antiviral-signaling protein knockdown, indicating a RIG-I-like receptor-independent function. In contrast, in a cell culture model of either poly-IC transfection or pattern recognition receptor overexpression, ADAP2 positively regulated IFN responses ^29^. Additional studies are needed to determine whether this phenotype is linked to ADAP2-induced macropinocytosis.

### DDX60L

In addition to RIG-I and MDA5, which are both DExD/H-box helicases, two other DEAD box helicases—DDX60 and the highly homologous DDX60L—are known ISGs. Previous studies have implicated a role for DDX60 in potentiating RIG-I-mediated signaling and suppressing HCV when overexpressed ^27, 30^. In a recent study, *DDX60L* was newly identified as a gene differentially expressed in response to IFNγ in Huh7 as compared with Huh6 hepatoma cells, the latter of which are refractory to IFNγ-mediated inhibition of HCV ^31^. DDX60L is required for full IFN-mediated inhibition of HCV in Huh7 cells and acts directly without affecting other ISG responses. Mechanistically, DDX60L suppressed HCV replication independently of effects on viral translation or genomic RNA degradation. Moreover, DDX60L potently suppressed lentivirus production, suggesting a broader antiviral function.

### SERPINE1

In a large-scale scale screen for ISGs that suppress late stages of influenza A virus infection, Dittmann *et al*. identified *SERPINE1* as a potent anti-influenza ISG ^32^. *SERPINE1* encodes plasminogen activator inhibitor 1 (PAI-1), which targets critical cellular proteases that are required for the cleavage of influenza A virus hemagglutinin and maturation of the viral particle. Thus, in the presence of PAI-1, newly formed influenza A virus particles are less infectious. Importantly, the authors demonstrated the relevance for this mechanism *in vivo*. *Serpine1* ^−/−^ mice were more susceptible to pathogenic outcomes of influenza A virus infection. Additionally, human cells from patients with reduced PAI-1 levels due to mutations in *SERPINE1* were more permissive to influenza A virus infection. This study highlights an unusual antiviral mechanism, in which an ISG effector functions outside the cell to inhibit a late stage of virus production.

## Perspectives

This review has largely highlighted effects of single ISGs that have been studied in reductionist approaches, typically by ectopic expression or by gene silencing. It is important to note that single ISG effects are generally difficult to uncover and to validate, particularly in relevant models of viral pathogenesis. This is likely due to the inherent redundancy in the IFN/ISG system. If, for example, multiple ISGs target a single virus, then studying one of those ISGs in isolation may confer only modest phenotypes with respect to virus yield or pathogenic outcomes. Thus, the in-depth study of a single ISG will benefit from careful selection of host cell type and virus in order to achieve a robust experimental system that affords that greatest level of protection.

Mouse models of single ISG knockouts have been critical for understanding the contribution of certain genes to antiviral protection *in vivo*. However, *in vivo* models in mice should not be the only litmus test to assess ISG relevance. Accumulating evidence suggests that ISGs may have species-specific differences. Alternatively, some ISGs, like human *IFI6*, do not have mouse orthologs. Thus, the assumption that a gene-specific knockout mouse is going to be the most relevant model for a human ISG should be met with caution. Of course, with the advent of rapid gene targeting in mice by clustered regularly interspaced short palindromic repeats (CRISPR), such hypotheses can be tested relatively quickly and inexpensively compared with older, time-consuming knockout strategies. In addition to standard gene targeting in mice, genetic studies in species-relevant primary cell cultures and organoid models may help ascribe ISG relevance to antiviral response pathways when mouse models will not suffice. Combining insight from relevant pathogenesis models with in-depth ISG mechanism of action studies may then lay a foundation for the development of therapeutic interventions that capitalize on antiviral ISG effector functions.
